# ^18^F-FDG positron emission tomography scanning in systemic sclerosis-associated interstitial lung disease: a pilot study

**DOI:** 10.1186/s13075-021-02460-8

**Published:** 2021-03-06

**Authors:** Emmanuel Ledoult, Maxime Morelle, Michael Soussan, Arsène Mékinian, Hélène Béhal, Vincent Sobanski, Eric Hachulla, Damien Huglo, Noémie Le Gouellec, Martine Remy-Jardin, Clio Baillet, David Launay

**Affiliations:** 1 Univ. Lille, INFINITE - Institute for Translational Research in Inflammation, F-59000 Lille, France; 2 Univ. Lille, CHU Lille, Service de Médecine Interne, Centre de Référence des Maladies Auto-immunes et Systémiques Rares du Nord et Nord-Ouest de France (CeRAINO), F-59000 Lille, France; 3 Inserm, U1286, F-59000 Lille, France; 4 Hôpital Claude Huriez, Service de Médecine Interne, Rue Michel Polonovski, F59037 Lille Cedex, France; 5 CHU Lille, Service de Médecine Nucléaire, F-59000 Lille, France; 6 CH Avicenne – APHP, Service de Médecine Nucléaire, F-93000 Bobigny, France; 7 Hôpital Saint-Antoine – APHP, Service de Médecine Interne, F-75012 Paris, France; 8 Sorbonne Université, F-75571 Paris Cedex 12, France; 9 Univ. Lille, CHU Lille, ULR 2694 - METRICS : Évaluation des technologies de santé et des pratiques médicales, F-59000 Lille, France; 10 Univ. Lille, Inserm, CHU Lille, U1189 - ONCO-THAI - Image Assisted Laser Therapy for Oncology, F-59000 Lille, France; 11 CH Valenciennes, Service de Médecine Interne, Centre de Compétences adultes pour les maladies auto-immunes et systémiques rares, F-59300 Valenciennes, France; 12 Univ. Lille, CHU Lille, Service d’imagerie Thoracique, F-59000 Lille, France; 13 Univ. Lille, CHU Lille, EA 7365 - GRITA - Groupe de Recherche sur les formes Injectables et les Technologies Associées, F-59000 Lille, France

**Keywords:** Systemic sclerosis, Interstitial lung disease, ^18^F-FDG PET/CT, Pulmonary function tests

## Abstract

**Background:**

Interstitial lung disease is a common complication of systemic sclerosis (SSc-ILD), and it remains difficult to accurately predict its course. Progressing ILD could be more metabolically active, suggesting that the ^18^F-FDG tracer could be a tool in the managing of SSc-ILD.

**Methods:**

In our center, SSc patients and controls (non-Hodgkin lymphoma cured after first-line regimen) who had received a PET/CT were screened retrospectively. The FDG uptake (visual intensity, pattern, SUV_max_) was systematically recorded in > 30 regions of interest (ROIs) linked to SSc in a blind reviewing by 2 independent nuclear medicine physicians using a standardized form.

**Results:**

Among the 545 SSc patients followed up in our center, 36, including 22 SSc-ILDs, had a PET/CT, whose indication was cancer screening in most cases. The mean ± SD age was 57.9 ± 13.0 years with 20/36 females. Fourteen patients had a disease duration of less than 2 years. A third had anti-centromere antibodies and 27.8% had anti-topoisomerase antibodies. Pulmonary FDG uptakes were higher in SSc patients than in controls (*n* = 89), especially in those with ILD compared with those without ILD. Pulmonary FDG uptakes were positively correlated with the ILD severity (fibrosis extent, %FVC, and %D_LCO_). No significant difference was found in the FDG uptakes from extrathoracic ROIs. Progressing SSc-ILDs within the 2 years after PET/CT (*n* = 9) had significant higher pulmonary FDG uptakes at baseline than stable SSc-ILDs (*n* = 13).

**Conclusion:**

PET/CT could be a useful tool in the assessment of the severity and the prediction of pulmonary function outcome of SSc-ILD.

**Supplementary Information:**

The online version contains supplementary material available at 10.1186/s13075-021-02460-8.

## Background

Interstitial lung disease (ILD) is a frequent complication in systemic sclerosis (SSc) [1, 2]. It is also the first cause of death attributable to SSc [3, 4]. The most common histopathologic findings are nonspecific interstitial pneumonia (NSIP; ~ 78%) [5]. Managing SSc-ILD is challenging because of the great heterogeneity of its courses, which can be stable or slowly or quickly deteriorating [6–8], and the modest benefit of immunosuppressants [9–12]. The initiation of immunosuppressants for SSc-ILD is usually recommended and more efficient in patients with active and/or progressing ILD, currently evaluated by the extension observed in high-resolution computed scans (HRCTs) and the deterioration in pulmonary function tests (PFTs; i.e., forced vital capacity [FVC] and diffusing capacity for the lung of carbon monoxide [D_LCO_]) [12]. Indeed, inflammation and the immune system could play a greater role in SSc patients with active and/or progressing ILD than in those with a stable course and could explain the effects of immunosuppressants in the first group of patients [13, 14]. However, it remains difficult to accurately predict ILD evolution [15–17]. Tools and biomarkers must be developed to assess ILD activity and the predictors of ILD progression and treatment response to optimize the decision-making [18, 19] in an era where, beyond immunosuppressants, new antifibrotic treatments, for example, nintedanib, are or will be available [20].

One hypothesis is that progressing or active ILD could be more metabolically active and suggests that (^18^F)-fluorodeoxyglucose (FDG), a tracer of glucose uptake and metabolism, could be useful. Nobashi et al. [21] provided evidence in a sample of 90 patients with ILD (of whom 51 were idiopathic NSIP) that there were significant but modest correlations between the mean of pulmonary standard uptake values (SUVs) and both %FVC and %D_LCO_, suggesting that FDG PET/CT could be a tool in ILD management. In 18 patients with cellular and fibrotic NSIP (1/18 had an SSc-ILD), Jacquelin et al. [22] reported increased pulmonary FDG uptake in areas of reticular (76%)/honeycombing (85%) and ground-glass opacity (89%) on HRCT, as well as in areas with a normal morphological appearance of the lung parenchyma on HRCT [23]. FDG PET/CT could therefore be more sensitive than HRCT alone in detecting early ILD and could allow early detection of therapeutic response. Together, these results suggest a need to provide more data on FDG PET/CT findings in SSc, especially in SSc-ILD [8]. Additionally, FDG PET/CT findings in extrapulmonary regions of interests (ROIs) linked to SSc have been scarce [24–26], and no study has reported systematic analysis of FDG extrapulmonary uptake related to SSc.

There are several challenges in the assessment of SSc-ILD using PET/CT because of the lack of a standardized approach; great variability of NSIP, which must be considered during the PET analysis process; and increased FDG uptake, which is not linked to SSc, for example, in the lung: neoplastic nodule, lung infection, or respiratory effects [27–30].

To address these issues, we systematically recorded the visual examination, the intensity, and the SUV_max_ of FDG uptake in > 30 ROIs related to SSc (pulmonary or extrapulmonary) on PET/CT scans among 36 SSc patients with or without ILD and 89 controls. We provided a detailed description of PET/CT findings, and they support that this tool could be useful in SSc, especially in SSc-ILD.

## Methods

### Systemic sclerosis patients and controls

This retrospective study was led in a French national SSc reference center. Participants had to be referred to have an FDG PET/CT performed between January 2011 and December 2017 and aged over 18 years. SSc patients had to fulfill the 1980-ACR Scleroderma [31] or 2013-ACR/EULAR SSc [32] classification criteria. Because there are no standardized reference values for pulmonary SUV, controls were retrospectively included among patients with non-Hodgkin lymphoma cured after first-line regimen to evaluate the physiological FDG uptake. Controls had to fulfill the following criteria: (i) aged over 18 years, (ii) no solid organ involvement in the history of lymphoma, (iii) no treatment received known for lung side effects such as bleomycin or mediastinal radiotherapy, (iv) in complete remission for 2 years, and (v) a non-pathologic FDG PET/CT at the time of inclusion. All controls were followed up in the hematologic department of the same university hospital. Exclusion criteria were lung infection and lung neoplasia, making uninterpretable the systematic collection of FDG uptakes because of potential interference. Patients with pulmonary nodules, which were outside the recorded areas, were not excluded. Data protection complied with the requirements of the National Information Science and Liberties Commission (CNIL number DEC18-355). For this study, formal consent was not required according to French legislation.

### Clinical data collection and variables

The inclusion was defined as the time of PET/CT. All clinical and biological data were collected in the history of the disease at the time of the inclusion by using a standardized form. Data collected were demographics, dates of first Raynaud phenomena (RP) and first non-RP sign, cutaneous subset [31], telangiectasia, calcinosis, dates and results of lung HRCT and PFTs, and autoantibody status (anti-centromere, anti-topoisomerase I, anti-RNA polymerase III, other autoantibodies). The disease duration was defined as the time elapsed between the first non-RP sign by the patient report and the inclusion visit. The organ involvements were defined by the occurrence of the following clinical events: (1) for joint: by arthritis, arthralgia, friction rubs, or synovitis; (2) for muscle: by myalgia, myositis, or rhabdomyolysis; (3) for SSc-ILD: by lung fibrosis on HRCT. SSc-ILD was classified as limited or extensive, based on lung fibrosis extent on HRCT with the use of %FVC according to Goh’s staging in intermediary cases [33]. ILD was considered as progressing if at least one of the following criteria for progression of ILD within the 2 years after PET/CT were met: (i) a relative decline in the %FVC of at least 10% of the predicted value, (ii) a relative decline in the %FVC of 5% to less than 10% of the predicted value and worsening of respiratory symptoms or/and an increased extent of fibrosis on HRCT [20]; (4) for the heart: by arrhythmia or conduction block or systolic dysfunction or pericardial effusion; (5) for pulmonary hypertension: confirmation by right heart catheterization; (6) for gastrointestinal tract (GIT): by reflux, dysmotility, constipation, diarrhea, signs of bacterial overgrowth and/or malabsorption; (7) for digital ulcer: by the past or current presence of digital ulcers or digital tips or pitting scars or digital ischemia; and (8) for renal crisis: by history of scleroderma renal crisis.

### FDG PET/CT scan analysis

FDG PET/CTs were performed on 4 different PET cameras (General Electric Discovery RX, *n* = 121; Siemens mCT FLOW, *n* = 5; General Electric Discovery 710, *n* = 2; General Electric Discovery Elite, *n* = 2). The mean ± SD times of post-injection were 73.5 ± 12.3 min and 73.5 ± 12.0 min after the intravenous injection of FDG in SSc patients and in controls, respectively. The analyzed FDG PET/CT areas ranged from the middle of the head to the middle of the thigh. At the time of injection, the mean capillary glycemia ± SD were 1.0 ± 0.3 and 1.0 ± 0.2 g/L in SSc patients and controls, respectively. A low-dose CT scan without iodine contrast agent was performed for attenuation correction, localization, and interpretation.

The ROIs were (1) lungs: each lung was split in 5 representative levels (the origin of the great vessels, the carina, the pulmonary venous confluence, at halfway between the third and fifth section, and 1 cm above the right dome of the diaphragm) according to the usual assessment of SSc-ILD, which represented 10 pulmonary ROIs [33, 34]; (2) muscles: deltoids, pectorals, rectus abdominis muscle, and quadriceps; (3) joints: gleno-humeral and coxo-femoral joints; (4) the skin: shoulders, breasts, peri-umbilical, and antero-superior part of the tights; (5) lymph nodes: hilar and mediastinal lymph nodes; and (6) the esophagus. For each ROI, the visual examination of FDG uptake was classified into a binary variable: nonpathological or abnormal pattern. The intensity of the FDG uptake was classified into 4 grades: grade = 0: no uptake; grade = 1: less intense than the liver; grade = 2: equally intense as the liver; and grade = 3: more intense than the liver. Measurements of SUV_max_ (normalized for the body weight) were also performed. Multilevel variables were recorded to evaluate both the intensity and the extent of the pulmonary FDG uptake: (i) the highest value among the 10 pulmonary SUV_max_ (hv/SUV_max_), (ii) the sum of the 10 pulmonary SUV_max_ (S/SUV_max_), and (iii) the sum of the 10 pulmonary visual intensities (S/Intensities). The FDG uptakes from the liver, mediastinum, because of respiratory effects or SSc-unlinked anomalies were excluded from the pulmonary areas of PET/CT scan analyses.

An independent review of all PET/CT scans was separately performed by 2 nuclear medicine physicians (seniors) using a standardized form. Both readers were blinded to clinical data and case/control status. The investigators received training sessions before the study. Interobserver variability was evaluated and determined to be satisfactory for consistency in visual examination and SUV_max_ measurements. Discrepancies were resolved during a consensus reading session.

### Statistical procedures

The continuous variables were expressed by the mean ± SD or the median with interquartile range (IQR). The normality of distribution was checked graphically and using the Shapiro–Wilk test. The categorical variables were expressed as frequencies and percentages. The comparisons were performed by using the *t* test or Mann–Whitney test for quantitative variables and the Fisher exact test or the chi-square test for categorical variables. The comparisons of lung FDG/PET parameters were adjusted on BMI by using an analysis of variance model for hv/SUV_max_, S/SUV_max_, and S/Intensities, and a logistic regression model for the nonpathological/abnormal lung pattern. The correlations between the FDG uptake and clinical variables were assessed using the Spearman correlation coefficient. All statistical tests were performed at the 2-tailed *α* level of 0.05, and data analysis was performed by using SAS software version 9.4 (SAS Institute Inc., Cary, NC, USA) and GraphPad Prism version 8.0.0 (GraphPad Software, San Diego, CA, USA).

## Results

### Participant characteristics

**Fig. 1:**
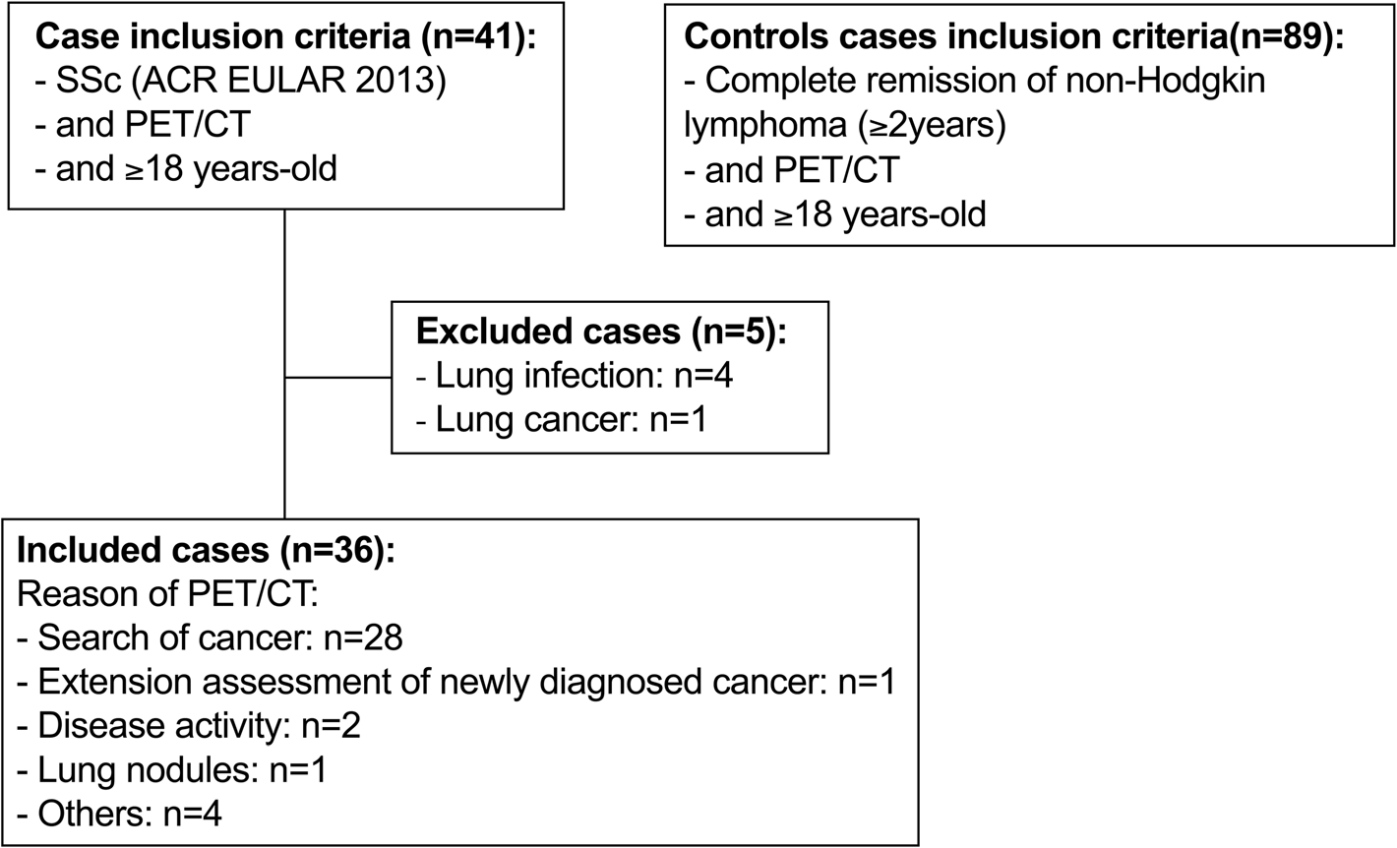
Flowchart. SSc systemic sclerosis

Of the 545 SSc patients followed in our center, 41 had performed an FDG PET/CT. Among these patients, those who had a clinically apparent infectious lung disease (*n* = 4) and those who had a lung primary or metastatic tumor (*n* = 1) that would confound measures of the lung ROIs were excluded. We thus retrospectively analyzed all the PET/CT scans from the 36 remaining SSc patients (20 females). The FDG PET/CTs were performed for the following reasons: search for a neoplasia (*n* = 28), evaluation of a pulmonary nodule (*n* = 1), new diagnosis of extrapulmonary cancer (*n* = 1), SSc activity (*n* = 2), suspicion of aortitis (*n* = 2), positive interferon-γ release assay (*n* = 1), and the diagnosis work-up before an autologous stem cell transplantation (*n* = 1; Fig. 1).

The mean ± SD age was 57.9 ± 13.0 years with a mean ± SD BMI of 25.3 ± 5.9 kg/m^2^. Among them, 14/36 had a disease duration of less than 2 years. Anti-nuclear antibodies were found in 34 patients including 12 with anti-centromere antibodies, 10 with anti-topoisomerase antibodies, and 4 with anti-RNA polymerase 3 antibodies. The half had a limited cutaneous subset. The median (IQR) mRSS was 12 (4.0; 21.0). Twenty-two patients had SSc-ILD (Table 1). To determine if FDG PET/CT scan findings were different in SSc patients and controls, we first analyzed blindly. Potential confusion factors were not significantly different between SSc patients and controls (Table 2).

**Table 1:** Characteristics of the SSc patients (*n* = 36)

	No. with available data	SSc patients (***n*** = 36)
**Demographics**		
Sex, female, no. (%)	36	20 (55.6)
Age, years, mean ± SD	36	57.9 ± 13.0
Ethnical, no. (%)	33	
White		30 (90.9)
Black		2 (6.1)
Asian		1 (3.1)
BMI, kg/m^2^, mean ± SD	36	25.3 ± 5.9
**Disease characteristics**		
Cutaneous subset, limited, no. (%)	36	19 (52.8)
Disease duration, no. (%)	36	
< 2 years		14 (38.9)
2–4 years		8 (22.2)
> 4 years		14 (38.9)
Antibody status, no. (%)	36	
Anti-nuclear		34 (94.4)
Anti-centromere		12 (33.3)
Anti-topoisomerase I		10 (27.8)
Anti-ARN polymerase III		4 (11.1)
**Organ involvement, no. (%)**		
Lung, no. (%)		
Interstitial lung disease	36	22 (61.1)
Current %FVC, median (IQR)	34	90.0 (80.0; 100.0)
Current %D_LCO_, median (IQR)	32	60.0 (40.0; 70.0)
Osteoarticular	32	14 (43.8)
Muscle	24	6 (25.0)
Current mRSS, median (IQR)	33	12 (4.0; 21.0)
Gastrointestinal tract	36	24 (66.7)
Pulmonary hypertension	36	8 (22.2)
Renal crisis	36	0 (0.0)
Calcinosis	33	4 (12.1)
Telangiectasia	35	22 (62.9)
Ulcer digital	34	10 (29.4)
Heart	36	6 (16.7)
**Current treatment**, no. (%)		
Steroids	36	13 (36.1)
Immunosuppressive drugs	36	13 (36.1)

*BMI* body mass index, *%D*_*LCO*_ diffusing capacity for the lung of carbon monoxide (% predicted value), *%FVC* forced vital capacity (% predicted value), *IQR* interquartile range, *SD* standard deviation, *SSc* systemic sclerosis

**Table 2:** Confusion factors and FDG PET/CT scan findings in controls (*n* = 89) and in SSc patients (*n* = 36)

	Controls (*n* = 89)	SSc patients (*n* = 36)	***p*** value
**Potential confusion factors**			
Sex, female, no. (%)	47 (52.8)	20 (55.6)	0.78
Age, years, mean ± SD	61.7 ± 12.8	57.9 ± 13.0	0.23
BMI, kg/m^2^, mean ± SD	25.9 ± 5.1	25.3 ± 5.9	0.59
Glycemia, g/L, mean ± SD	1.0 ± 0.3	1.0 ± 0.2	0.42
Injection-perfusion duration, minutes, mean ± SD	73.5 ± 12.3	73.5 ± 12.0	1.0
^**18**^**F-FDG PET/CT scan findings, no. (%)**			
Lung			
Abnormal interstitial lung pattern	1 (1.1)	15 (41.7)	<.001^†^
hv/SUV_max_, mean ± SD	1.5 ± 0.3	2.1 ± 1.0	<.001^†^
S/SUV_max_, mean ± SD	11.1 ± 2.2	14.4 ± 6.4	0.005^†^
S/Intensities, median (IQR)	0.0 (0.0; 0.0)	2.5 (0.0; 10.0)	<.001^†^
Lymph node			
Mediastinal, abnormal pattern	0 (0.0)	9 (25.0)	<.001
Hilar, abnormal pattern	2 (2.2)	8 (22.2)	<.001
Osteoarticular, abnormal pattern	2 (2.2)	1 (2.8)	NA
Muscles, abnormal pattern	2 (2.2)	3 (8.3)	NA
Skin, abnormal pattern	2 (2.2)	2 (5.6)	NA
Esophagus, abnormal pattern	81 (91.0)	26 (72.2)	0.007
Calcinosis, abnormal pattern	0 (0.0)	0 (0.0)	NA

*BMI* body mass index, *hv/SUV*_*max*_ highest value of SUV_max_ among the 10 pulmonary SUV_max_, *IQR* interquartile range, *NA* non-applicable, *SD* standard deviation, *S/Intensities* sum of the 10 pulmonary intensities, *S/SUV*_*max*_ sum of the 10 pulmonary SUV_max_, *SSc* systemic sclerosis. ^†^*p* value adjusted on BMI, <.001

### FDG PET/CT scan findings for pulmonary multilevel ROIs

#### In SSc patients versus controls

Considering both the visual examination and semi-quantitative measurements, FDG PET/CT scans revealed higher pulmonary FDG uptakes in SSc patients than in controls: more interstitial lung abnormal patterns (41.7% vs. 1.1%, *p* < .001), a higher mean hv/SUV_max_ (2.1 ± 1.0 vs. 1.5 ± 0.3, *p* < .001), a higher mean S/SUV_max_ (14.4 ± 6.4 vs. 11.1 ± 2.2, *p* = 0.005), and a higher median S/Intensities (2.5 [0.0; 10.0] vs. 0.0 [0.0; 0.0], *p* < .001). These results were similar using BMI as an adjustment factor (Table 2).

#### In SSc patients with ILD versus those without ILD

**Fig. 2:**
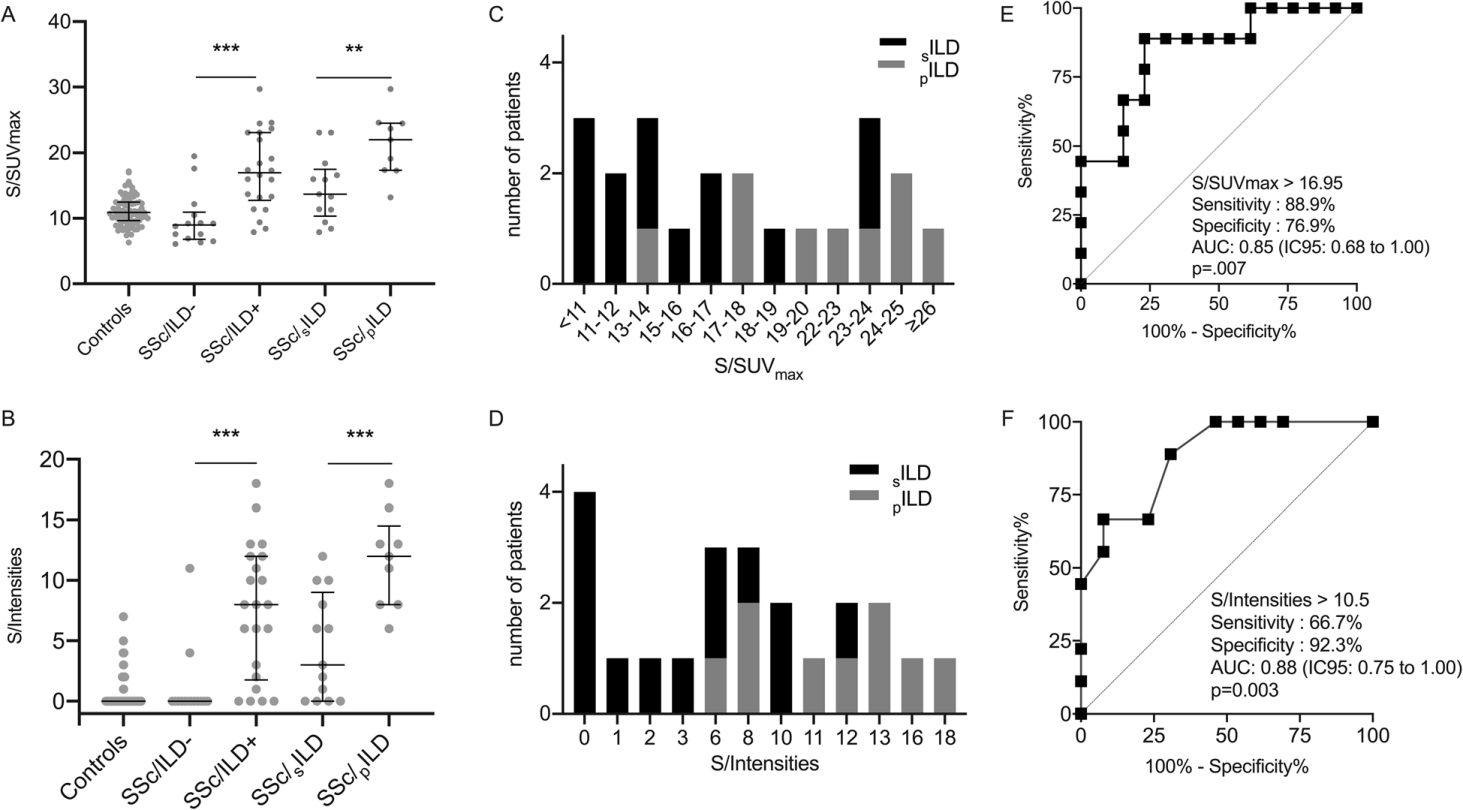
Pulmonary FDG uptake. **a**, **b** S/SUV_max_ and S/Intensities in controls (*n* = 89), in SSc patients without ILD (*n* = 14), in SSc patients with ILD (*n* = 22), in SSc patients with stable ILD (*n* = 13), and in SSc patients with progressing ILD (*n* = 9). **c**, **d** Number of stable ILD and progressing ILD per baseline S/SUV_max_ and S/Intensities ranges. **e**, **f** The ROC curve analysis of S/SUV_max_ and S/Intensities for the prediction of progressing ILD. Data are shown as the median with interquartile range. _s_ILD, stable interstitial lung disease; _p_ILD, progressing interstitial lung disease; SSc, systemic sclerosis. *p < 0.05; **p < 0.01; ***p < 0.001

**Fig. 3:**
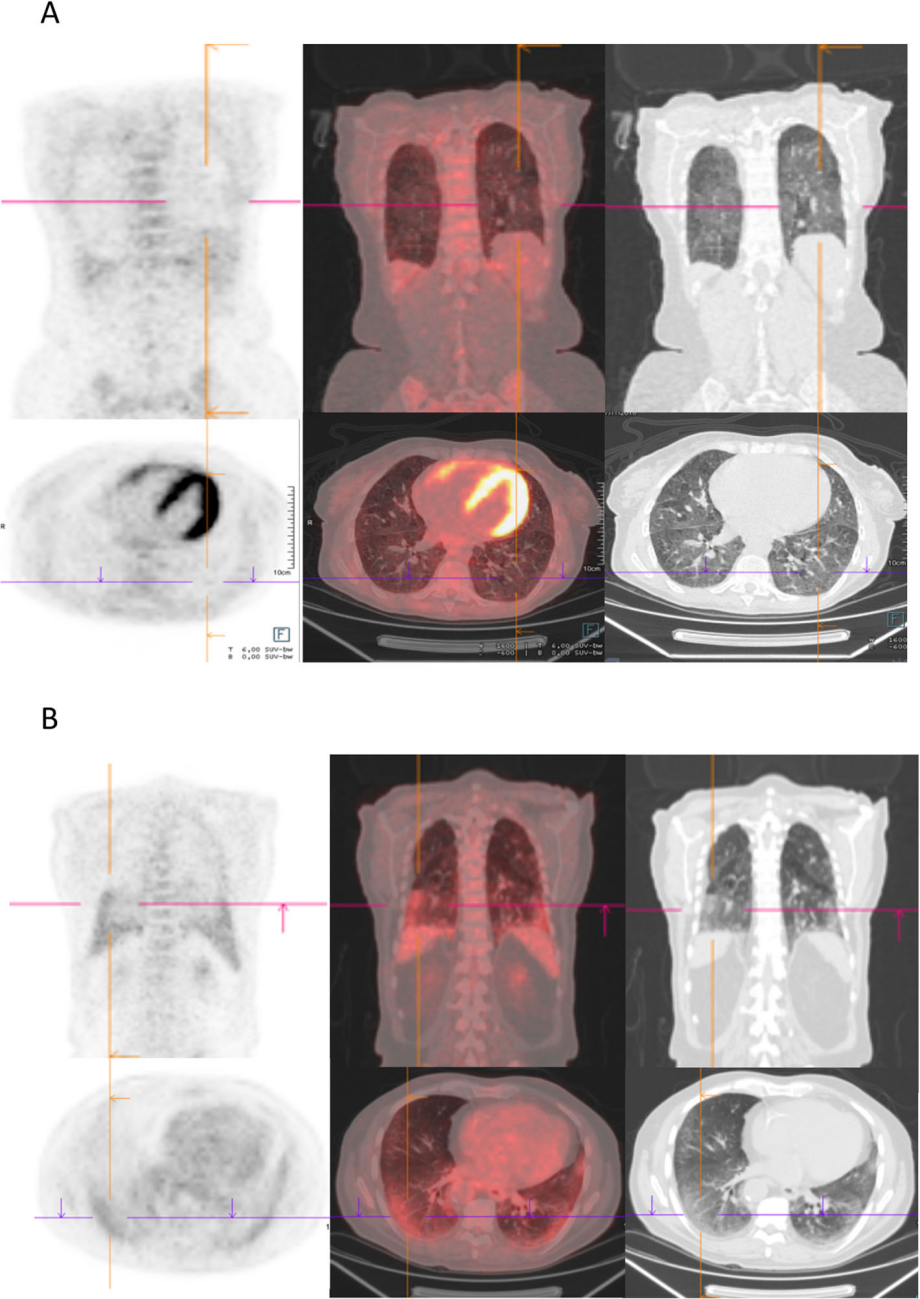
Visual examination of FDG PET/CT scans. Transverse slides of the FDG PET/CT scans of **a** a SSc-ILD with an abnormal interstitial lung pattern of FDG uptake and **b** a SSc-ILD without an abnormal interstitial lung pattern of FDG uptake

SSc patients with ILD (*n* = 22) were mainly (59.1%) males and had a mean ± SD age of 61.3 ± 10.6 years, while SSc patients without ILD (*n* = 14) were mainly females (78.6%, *p* = 0.027) and had a mean age of 54.6 ± 14.3 years (*p* = 0.12). Anti-topoisomerase antibodies were found in 9/22 SSc patients with ILD and in 1/14 SSc patients without ILD (*p* = 0.054). The median disease duration of ILD was 2.0 (0.0; 8.5) years at inclusion. Among SSc-ILD patients, 13/22 had an extensive ILD according to Goh’s classification. The median lung fibrosis extent on HRCT was 50.0% (10.0; 80.0) (Additional file 1). The pulmonary FDG uptakes were higher in SSc patients with ILD than in those without ILD: more interstitial lung abnormal patterns (14/22 vs. 1/14, *p* < .001), a higher mean hv/SUV_max_ (2.6 ± 0.9 vs. 1.4 ± 0.8, *p* < .001), a higher mean S/SUV_max_ (17.3 ± 6.0 vs. 9.8 ± 4.1, *p* < .001), and a higher median S/Intensities (8.0 [2.0; 12.0] vs. 0.0 [0.0; 0.0], *p* < .001). These results were similar when using BMI as an adjustment factor (Table 3, Figs. 2 and 3).

**Table 3:** Characteristics and FDG PET/CT scan findings in SSc patients without ILD (*n* = 14) and with ILD (*n* = 22)

	SSc patients without ILD (***n*** = 14)	SSc patients with ILD (***n*** = 22)	***p*** value
**Potential confusion factors**			
Sex, female, no. (%)	11 (78.6)	9 (40.9)	0.027
Age, years, mean ± SD	54.6 ± 14.3	61.3 ± 10.6	0.12
BMI, kg/m^2^, mean ± SD	25.6 ± 5.8	25.1 ± 6.0	0.80
Glycemia, g/L, mean ± SD	1.1 ± 0.3	1.0 ± 0.1	0.35
Injection-perfusion duration, minutes, mean ± SD	70.3 ± 11.9	75.6 ± 11.9	0.20
**Disease characteristics**			
Cutaneous subset, limited, no. (%)	8 (57.1)	11 (50.0)	0.68
Disease duration, no. (%)			0.11
< 2 years	8 (57.1)	6 (27.3)	
2–4 years	1 (7.1)	7 (31.8)	
> 4 years	5 (35.7)	9 (40.9)	
Antibody status, no. (%)			
Anti-nuclear	13 (92.9)	21 (95.5)	NA
Anti-centromere	7 (50.0)	5 (22.7)	0.15
Anti-topoisomerase I	1 (7.1)	9 (40.9)	0.054
Anti-RNA polymerase III	1 (7.1)	3 (13.6)	NA
**Organ involvement, no. (%)**			
Lung			
Current %FVC, mean ± SD	100 ± 10	80 ± 20	0.015
Current %D_LCO_, mean ± SD	70 ± 20	50 ± 20	0.002
Osteoarticular	7 (58.3)	7 (35.0)	0.20
Muscle	3 (27.3)	3 (23.1)	NA
Current mRSS, mean ± SD	13.6 ± 13.1	13.9 ± 11.5	0.96
Gastrointestinal tract	9 (64.3)	15 (68.2)	1.00
Pulmonary hypertension	3 (21.4)	5 (22.7)	NA
Calcinosis	2 (14.3)	2 (10.5)	NA
Telangiectasia	11 (78.6)	11 (52.4)	0.12
Ulcer digital	3 (21.4)	7 (35.0)	0.47
Heart	1 (7.1)	5 (22.7)	NA
**Current treatments, no. (%)**			
Steroids	3 (21.4)	10 (45.5)	0.14
Immunosuppressive drugs	3 (21.4)	10 (45.5)	0.14
^**18**^**F-FDG PET/CT scan findings, no (%)**			
Lung			
Abnormal interstitial pattern	1 (7.1)	14 (63.6)	<.001^‡^
hv/SUV_max_, mean ± SD	1.4 ± 0.8	2.6 ± 0.9	<.001^†^
s/SUV_max_, mean ± SD	9.8 ± 4.1	17.3 ± 6.0	<.001^†^
s/Intensities, median (IQR)	0.0 (0.0; 0.0)	8.0 (2.0; 12.0)	<.001^†^
Lymph node			
Mediastinal, abnormal pattern	2 (14.3)	7 (31.8)	0.43
Hilar, abnormal pattern	2 (14.3)	6 (27.3)	0.44
Osteoarticular, abnormal pattern	0 (0.0)	1 (4.5)	NA
Muscle, abnormal pattern	1 (7.1)	2 (9.1)	NA
Skin, abnormal pattern	1 (7.1)	1 (4.5)	NA
Esophagus, abnormal pattern	9 (64.3)	17 (77.3)	1.0

*BMI* body mass index, *%FVC* forced vital capacity (as % predicted values), *%D*_*LCO*_ diffusing capacity for the lung of carbon monoxide (as % predicted values), *hv/SUV*_*max*_ highest value of the SUV_max_ among the 10 pulmonary SUV_max_, *IQR* interquartile range, *SD* standard deviation, *s/Intensities* sum of the 10 pulmonary intensities, *s/SUV*_*max*_ sum of the 10 pulmonary SUV_max_, *NA* not applicable. ^‡^*p* value adjusted on BMI = 0.021; ^†^*p* value adjusted on BMI, <.001

#### Correlations between ILD parameters and FDG PET/CT findings

We found significant albeit modest correlations between (i) the S/SUVmax with %DLCO (*r* = − 0.494, *p* = 0.004) and with fibrosis extent (*r* = 0.475, *p* = 0.026), (ii) the S/Intensities with %FVC (*r* = − 0.426, *p* = 0.012) and with %DLCO (*r* = − 0.484, *p* = 0.004), and (iii) the hv/SUVmax with %DLCO (*r* = − 0.438, *p* = 0.011; Additional file 2). We found no significant difference in the pulmonary FDG uptakes (SUVmax and intensity) according to the morphological modifications on representative levels. Honeycombing was not detected in the ROIs. We also did not find a significant difference in the pulmonary FDG uptakes between patients with limited ILD and those with extensive ILD (Additional file 3).

#### In SSc patients with progressing ILD versus those with stable ILD

For exploratory purposes, we compared SSc patients according to the progressing ILD status within the 2 years after PET/CT. Nine cases of 22 SSc-ILD patients fulfilled the criteria of progressing ILD (_p_ILD, as defined in methods). At inclusion, the mean %FVC were 77 ± 22% and 92 ± 17%, and the mean %D_LCO_ were 45 ± 20% and 53 ± 16%, in _p_ILD and _s_ILD respectively. Within a respective median follow-up of 25.6 (14.1; 27.0) and 22.9 (17.8; 29.0) months after PET/CT, the mean change of %FVC was − 11% (− 6; − 14) in _p_ILD and + 4% (+ 1; + 8) in _s_ILD. The median S/Intensities was higher in _p_ILD (12.0 [8.0; 13.0]) than in _s_ILD (3.0 [0.0; 8.0], *p* < .001). The mean S/SUVmax were 21.3 ± 5.0 and 14.5 ± 5.0, respectively in _p_ILD and _s_ILD (*p* = 0.005). The ROC curves showed that the sensitivity and specificity of S/Intensities were 66.7% and 92.3% respectively (AUC 0.88, *p* = 0.003) with a cutoff value of 10.5, and they were 88.9% and 76.9% (AUC: 0.85, *p* = 0.007) with a cutoff value of 16.95 of S/SUV_max,_ for the progression of ILD, respectively (Figs. 2 and 3, Additional file 4). There was a case with limited ILD at inclusion becoming extensive 2 years later, who had a S/Intensities of 11.0 which was above the cutoff value of 10.5 suggested by the curve ROC for progressing ILD.

### FDG PET/CT scan findings for extrapulmonary ROIs

The visual examination and the systematic measurements of SUV_max_ for the ROIs are available in Table 2 and in Additional files 5 and 6. The visual examination of FDG uptakes revealed fewer abnormal uptake patterns for the esophagus in SSc patients than controls (*p* = 0.007). Among the 3 patients with abnormal patterns for the explored muscles, clinical muscle involvement was known for 1. In 2 SSc patients, the visual examination revealed abnormal patterns for the skin. mRSS were 13 and 14 respectively.

### Screening for hypermetabolism suspected of malignancy

FDG PET/CT scans revealed 13 hypermetabolism equivocal or suspect for malignancy in 10/36 SSc patients. Suspected foci were 5 digestive foci, 2 pulmonary nodules, and 1 breast focus. Equivocal foci were 1 thyroid focus and 4 lymph node foci. Malignancy was confirmed in 3 SSc patients: 1 patient with ILD had a lung cancer, and 2 patients without ILD had a breast cancer and a digestive cancer, respectively.

## Discussion

According to our review of the literature, this study investigated the largest series of SSc patients by using PET/CT to assess pulmonary FDG uptake and the first to explore extrapulmonary PET findings. The pulmonary FDG uptake was higher in SSc patients than in controls and especially in patients with SSc-ILD than in those without SSc-ILD. The pulmonary FDG uptake was also positively correlated with the ILD severity.

Our results are in line with experimental and clinical studies. In bleomycin-induced pulmonary fibrosis in mice, FDG uptake increased early during the inflammatory stage and persisted in the later fibrotic stage. By combined FDG and FBEM–radiolabeled leukocytes, the authors showed that the early increase of FDG uptake was probably related to the early recruitment of leukocytes at the inflammatory stage (day 8), whereas the persistent elevation of FDG uptake at the fibrotic stage (day 15) was not explained by an active recruitment of leukocytes [35]. This increased FDG uptake at the fibrotic stage could be related to the aerobic glycolysis that has been demonstrated to be increased in myofibroblasts [36, 37]. In the same murine model, pirfenidone significantly reduced FDG uptake in the lungs during the fibrotic stage but not during the early inflammatory stage, suggesting that FDG PET/CT could assess the activity of the disease at the early stage. For exploratory purposes, we have distinguished _p_ILD, associated with a more probable inflammatory component, from _s_ILD within the 2 years after PET/CT. We found higher values of FDG uptake in _p_ILD than in _s_ILD, suggesting that FDG uptake could be useful to assess the severity and to predict the pulmonary function outcome of SSc-ILD. Importantly, these results are preliminary and that the very small number of patients did not allow us to adjust *p* values. Thus, this hypothesis should be explored in a larger study.

In humans, FDG PET/CT has been employed in ILD related to IPF [19, 29, 38]. Most studies have reported significant correlations between FDG uptake and parameters such as %FVC, %D_LCO_, and fibrosis extent, mostly in IPF [39–43]. However, IPF and SSc-ILD display different histological findings and disease courses, which could limit the scope of these results [5, 44]. Few small studies have focused on patients with SSc-ILD [45, 46]. As in Peelen et al. [45] who compared the FDG PET/CT scan findings from 8 SSc patients (3 had ILD), we founded increased FDG uptake in ground-glass opacities and reticulations. We also showed that the pulmonary FDG uptakes were higher in SSc patients with ILD than in those without ILD or in controls. Furthermore, we also found modest correlations between FDG uptake and %FVC, %D_LCO_, and fibrosis extent. Predictive values of FDG PET/CT scan findings have been already suggested in IPF. Umeda et al. [40] reported that the positive retention index of SUV, calculated from SUV obtained after 1-h (early SUV) and 3-h (delayed SUV) imaging in 50 patients, strongly predicted early deterioration of pulmonary function and high mortality in patients with IPF. In our study, one dcSSc patient with anti-RNA polymerase III antibodies (mRSS = 30/51) had an abnormal interstitial lung pattern of FDG uptake without ILD on HRCT. This patient developed later an SSc-ILD (Additional file 7). Win et al. [23] reported increased pulmonary FDG uptake in areas with a normal morphological appearance of the lung parenchyma on HRCT in IPF. These data encourage further exploration of the FDG PET/CT contribution in the management of SSc-ILD.

This study has several strengths and limitations. The first strength is that we provided a comprehensive analysis that included apparently healthy areas. Second, we described significant associations between multilevel parameters and the severity/activity of SSc-ILD, providing motivation to further evaluate this tool in SSc-ILD by using prospective data. Third, we employed several precautions in the PET analyses, for example, training sessions for the nuclear medicine physicians, a large cohort of controls, blind rereviewing, considering potential confounding factors of FDG uptake recording, and exclusion of areas of suspicious foci or respiratory effects.

The methodological limitations are mainly related to the single-center design and the inclusion of a relatively small number of patients, although this is the largest cohort reporting SSc patients with FDG PET/CT [45, 46]. There is potential bias due to the inclusion criteria. Indeed, most of the SSc patients had an FDG PET/CT as an assessment for possible neoplasia. Third, PET/CTs were performed on 4 types of PET cameras, which might cause bias. The small number of PETs performed on cameras 2 to 4 did not allow the PET camera to be used as an adjustment factor. Fourth, the analyzed PET/CT areas excluded the extremities of the limbs beyond the arms and thighs, limiting the scope of our results because the articular and skin involvements in SSc affect preferably these areas. Only a prospective FDG PET/CT including these areas could properly assess them. Due to our small sample size, we cannot exclude that several associations were overlooked because of a lack of adequate statistical power. Furthermore, we cannot exclude false-positive association regarding the multiple comparison issue. For these reasons, our results should be interpreted with caution and further studies are warranted to confirm our findings. Finally, we limited this study to the quantification of the SUV_max_ and the FDG uptake intensities. Other variables, for example, metabolic lung volume and total lesion glycolysis, could offer a better description of the FDG uptake in fibrotic areas [47].

## Conclusion

In this study, we suggest that FDG PET/CT could be an interesting tool in the assessment of the severity and the prediction of pulmonary function outcome of SSc-ILD. Further prospective studies are mandatory to confirm these results.

## Supplementary Information

**Additional file 1 **Additional characteristics of SSc patients with ILD (*n* = 22).

**Additional file 2 **Correlation between FDG PET/CT scan findings, lung fibrosis extent (%), %D_LCO_ and %FVC (*n* = 36).

**Additional file 3 **Characteristics and FDG PET/CT scan findings in SSc patients with limited ILD (*n* = 9) or with extensive ILD (*n* = 13).

**Additional file 4.** Characteristics and FDG PET/CT scan findings in SSc patients with stable ILD (n = 13) or progressing ILD (n = 9) within 2 years after PET/CT.

**Additional file 5 **Additional FDG PET/CT scan findings in controls (*n* = 89) and in SSc patients (n = 36).

**Additional file 6 **Additional FDG PET/CT scan findings in SSc patients without ILD (*n* = 14) and those with ILD (n = 22).

**Additional file 7.** Visual examination of FDG PET/CT scans of a case report.

## Data Availability

The data that support the findings of this study are available on separate scientific request (contact Prof. David Launay, Department of Internal Medicine, University of Lille, Lille, France; david.launay@chru-lille.fr), but restrictions apply to the availability of these data, which were used under license for the current study and, hence, are not publicly available.

## Abbreviations

BMI: Body mass index
D_LCO_: Diffusing capacity for the lung of carbon monoxide
FDG: 2-Deoxy-2-(18F) fluoro-D-glucose
HRCT: High-resolution computed scan
FVC: Forced vital capacity
ILD: Interstitial lung disease
_p_ILD: Progressing interstitial lung disease
_s_ILD: Stable interstitial lung disease
IQR: Interquartile range
PET: Positron emission tomography
PFT: Pulmonary function test
ROI: Region of interest
SD: Standard deviation
SSc: Systemic sclerosis
SUV: Standard uptake value
S/SUV: Sum of the 10 pulmonary SUV_max_
hv/SUV_max_: Highest value of SUV_max_ among the 10 pulmonary SUV_max_
S/Intensities: Sum of the 10 intensities (range 0–3) of the 10 pulmonary slides

## References

[1] Hachulla E, Launay D (2011). Diagnosis and classification of systemic sclerosis. Clin Rev Allergy Immunol.

[2] Le Pavec J, Launay D, Mathai SC, Hassoun PM, Humbert M (2011). Scleroderma lung disease. Clin Rev Allergy Immunol.

[3] Steen VD, Medsger TA (2007). Changes in causes of death in systemic sclerosis, 1972-2002. Ann Rheum Dis.

[4] Pokeerbux MR, Giovannelli J, Dauchet L, Mouthon L, Agard C, Lega J-C (2019). Survival and prognosis factors in systemic sclerosis: data of a French multicenter cohort, systematic review, and meta-analysis of the literature. Arthritis Res Ther.

[5] Bouros D, Wells AU, Nicholson AG, Colby TV, Polychronopoulos V, Pantelidis P (2002). Histopathologic subsets of fibrosing alveolitis in patients with systemic sclerosis and their relationship to outcome. Am J Respir Crit Care Med.

[6] Hoffmann Vold AM, Aaløkken TM, Lund MB, Garen T, Midtvedt Ø, Brunborg C (2015). Predictive value of serial high-resolution computed tomography analyses and concurrent lung function tests in systemic sclerosis. Arthritis Rheumatol.

[7] Man A, Davidyock T, Ferguson LT, Ieong M, Zhang Y, Simms RW (2015). Changes in forced vital capacity over time in systemic sclerosis: application of group-based trajectory modelling. Rheumatology (Oxford).

[8] Fischer A, Patel NM, Volkmann ER (2019). Interstitial lung disease in systemic sclerosis: focus on early detection and intervention. Open Access Rheumatol.

[9] Hoyles RK, Ellis RW, Wellsbury J, Lees B, Newlands P, Goh NSL (2006). A multicenter, prospective, randomized, double-blind, placebo-controlled trial of corticosteroids and intravenous cyclophosphamide followed by oral azathioprine for the treatment of pulmonary fibrosis in scleroderma. Arthritis Rheum.

[10] Tashkin DP, Elashoff R, Clements PJ, Goldin J, Roth MD, Furst DE (2006). Cyclophosphamide versus placebo in scleroderma lung disease. N Engl J Med.

[11] Volkmann ER, Tashkin DP, Li N, Roth MD, Khanna D, Hoffmann Vold AM (2017). Mycophenolate mofetil versus placebo for systemic sclerosis-related interstitial lung disease: an analysis of scleroderma lung studies I and II. Arthritis Rheumatol.

[12] Kowal-Bielecka O, Fransen J, Avouac J, Becker M, Kulak A, Allanore Y (2017). Update of EULAR recommendations for the treatment of systemic sclerosis. Ann Rheum Dis.

[13] Bérezné A, Ranque B, Valeyre D, Brauner M, Allanore Y, Launay D (2008). Therapeutic strategy combining intravenous cyclophosphamide followed by oral azathioprine to treat worsening interstitial lung disease associated with systemic sclerosis: a retrospective multicenter open-label study. J Rheumatol.

[14] Launay D, Buchdahl A-L, Bérezné A, Hatron P-Y, Hachulla E, Mouthon L (2018). Mycophenolate mofetil following cyclophosphamide in worsening systemic sclerosis-associated interstitial lung disease. J Scleroderma Relat Disord.

[15] Khanna D, Nagaraja V, Tseng C-H, Abtin F, Suh R, Kim G (2015). Predictors of lung function decline in scleroderma-related interstitial lung disease based on high-resolution computed tomography: implications for cohort enrichment in systemic sclerosis-associated interstitial lung disease trials. Arthritis Res Ther.

[16] Winstone TA, Assayag D, Wilcox PG, Dunne JV, Hague CJ, Leipsic J (2014). Predictors of mortality and progression in scleroderma-associated interstitial lung disease: a systematic review. Chest.

[17] Le Gouellec N, Duhamel A, Perez T, Hachulla A-L, Sobanski V, Faivre J-B (2017). Predictors of lung function test severity and outcome in systemic sclerosis-associated interstitial lung disease. Plos One.

[18] Skaug B, Assassi S (2019). Biomarkers in systemic sclerosis. Curr Opin Rheumatol.

[19] Weatherley ND, Eaden JA, Stewart NJ, Bartholmai BJ, Swift AJ, Bianchi SM (2019). Experimental and quantitative imaging techniques in interstitial lung disease. Thorax.

[20] Distler O, Highland KB, Gahlemann M, Azuma A, Fischer A, Mayes MD (2019). Nintedanib for systemic sclerosis–associated interstitial lung disease. N Engl J Med.

[21] Nobashi T, Kubo T, Nakamoto Y, Handa T, Koyasu S, Ishimori T (2016). FDG uptake in less affected lung field provides prognostic stratification in patients with interstitial lung disease. J Nucl Med.

[22] Jacquelin V, Mekinian A, Brillet PY, Nunes H, Fain O, Valeyre D (2016). FDG-PET/CT in the prediction of pulmonary function improvement in nonspecific interstitial pneumonia. A pilot study. Eur J Radiol.

[23] Win T, Thomas BA, Lambrou T, Hutton BF, Screaton NJ, Porter JC (2014). Areas of normal pulmonary parenchyma on HRCT exhibit increased FDG PET signal in IPF patients. Eur J Nucl Med Mol Imaging.

[24] Redureau E, Lairez O, Hitzel A, Pugnet G (2017). Can positron emission tomography be useful to manage systemic sclerosis cardiac involvement?. J Nucl Cardiol.

[25] Vadrucci M, Castellani M, Benti R (2016). Active subcutaneous calcinosis demonstrated by fluorine-18 fluorodeoxyglucose positron emission tomography/computed tomography in a case of limited. Indian J Nucl Med.

[26] Oksuzoglu K, Ozen G, Inanir S, Direskeneli RH (2015). Flip-flop phenomenon in systemic sclerosis on fluorodeoxyglucose positron emission tomography/computed tomography. Indian J Nucl Med.

[27] Travis WD, Costabel U, Hansell DM, King TE, Lynch DA, Nicholson AG (2013). An official American Thoracic Society/European Respiratory Society statement: update of the international multidisciplinary classification of the idiopathic interstitial pneumonias. Am J Respir Crit Care Med.

[28] Holman BF, Cuplov V, Millner L, Hutton BF, Maher TM, Groves AM (2015). Improved correction for the tissue fraction effect in lung PET/CT imaging. Phys Med Biol.

[29] Chen DL, Cheriyan J, Chilvers ER, Choudhury G, Coello C, Connell M (2017). Quantification of lung PET images: challenges and opportunities. J Nucl Med.

[30] Nambu A, Ozawa K, Kobayashi N, Tago M (2014). Imaging of community-acquired pneumonia: roles of imaging examinations, imaging diagnosis of specific pathogens and discrimination from noninfectious diseases. World J Radiol.

[31] LeRoy EC, Medsger TA (2001). Criteria for the classification of early systemic sclerosis. J Rheumatol.

[32] Hoogen F, Khanna D, Fransen J, Johnson SR, Baron M, Tyndall A (2013). 2013 classification criteria for systemic sclerosis: an American College of Rheumatology/European League Against Rheumatism Collaborative Initiative. Arthritis Rheum.

[33] Goh NSL, Desai SR, Veeraraghavan S, Hansell DM, Copley SJ, Maher TM (2008). Interstitial lung disease in systemic sclerosis: a simple staging system. Am J Respir Crit Care Med.

[34] Wells AU (2008). High-resolution computed tomography and scleroderma lung disease. Rheumatology (Oxford).

[35] Bondue B, Sherer F, Van Simaeys G, Doumont G, Egrise D, Yakoub Y (2015). PET/CT with 18F-FDG- and 18F-FBEM-labeled leukocytes for metabolic activity and leukocyte recruitment monitoring in a mouse model of pulmonary fibrosis. J Nucl Med.

[36] Xie N, Tan Z, Banerjee S, Cui H, Ge J, Liu R-M (2015). Glycolytic reprogramming in myofibroblast differentiation and lung fibrosis. Am J Respir Crit Care Med.

[37] Maher TM (2015). Aerobic glycolysis and the Warburg effect. An unexplored realm in the search for fibrosis therapies?. Am J Respir Crit Care Med.

[38] Chen DL, Schiebler ML, Goo JM, van Beek EJR (2017). PET imaging approaches for inflammatory lung diseases: current concepts and future directions. Eur J Radiol.

[39] Justet A, Laurent-Bellue A, Thabut G, Dieudonné A, Debray M-P, Borie R (2017). [18F] FDG PET/CT predicts progression-free survival in patients with idiopathic pulmonary fibrosis. Respir Res.

[40] Umeda Y, Demura Y, Morikawa M, Anzai M, Kadowaki M, Ameshima S (2015). Prognostic value of dual-time-point 18F-FDG PET for idiopathic pulmonary fibrosis. J Nucl Med.

[41] Lee EYP, Wong CS, Fung SL, Yan PK, Ho JCM (2014). SUV as an adjunct in evaluating disease activity in idiopathic pulmonary fibrosis - a pilot study. Nucl Med Commun.

[42] Win T, Lambrou T, Hutton BF, Kayani I, Screaton NJ, Porter JC (2012). 18F-Fluorodeoxyglucose positron emission tomography pulmonary imaging in idiopathic pulmonary fibrosis is reproducible: implications for future clinical trials. Eur J Nucl Med Mol Imaging.

[43] Umeda Y, Demura Y, Ishizaki T, Ameshima S, Miyamori I, Saito Y (2009). Dual-time-point 18F-FDG PET imaging for diagnosis of disease type and disease activity in patients with idiopathic interstitial pneumonia. Eur J Nucl Med Mol Imaging.

[44] Khanna D, Tashkin DP, Denton CP, Renzoni EA, Desai SR, Varga J. Aetiology, risk factors, and biomarkers in systemic sclerosis with interstitial lung disease. Am J Respir Crit Care Med. 2019. 10.1164/rccm.201903-0563CI. 10.1164/rccm.201903-0563CI PMC7068837 31841044

[45] Peelen DM, Zwezerijnen BGJC, Nossent EJ, Meijboom LJ, Hoekstra OS, Van der Laken CJ, et al. The quantitative assessment of interstitial lung disease with positron emission tomography scanning in systemic sclerosis patients. Rheumatology (Oxford). 2019. 10.1093/rheumatology/kez483. 10.1093/rheumatology/kez483 PMC7244784 31642912

[46] Bellando-Randone S, Tartarelli L, Cavigli E, Tofani L, Bruni C, Lepri G (2019). 18F-fluorodeoxyglucose positron-emission tomography/CT and lung involvement in systemic sclerosis. Ann Rheum Dis.

[47] Larson SM, Erdi Y, Akhurst T, Mazumdar M, Macapinlac HA, Finn RD (1999). Tumor treatment response based on visual and quantitative changes in global tumor glycolysis using PET-FDG imaging. The visual response score and the change in total lesion glycolysis. Clin Positron Imaging.

