# Supplementary material for: 18F-FDG positron emission tomography scanning in systemic sclerosis-associated interstitial lung disease: a pilot study
Source: Arthritis Res Ther. 2021 Mar 6;23:76. doi: 10.1186/s13075-021-02460-8 (PMC7936499; doi:10.1186/s13075-021-02460-8)
Supplement: Supplementary file 1 — Additional file 1 Additional characteristics of SSc patients with ILD (n = 22). [file 13075_2021_2460_MOESM1_ESM.docx]

**Additional file 1.** Additional characteristics of SSc patients with ILD (n=22)

|  | **No. with available data** | **SSc with ILD**  **(n=22)** |
| --- | --- | --- |
| **Disease characteristics** |  |  |
| ILD duration, median (IQR), years | 22 | 2.0 (0.0; 8.5) |
| **HRCT results** |  |  |
| Duration from PET, median (IQR), months | 22 | 0.0 (-1.0; 1.0) |
| Ground glass, no. (%) | 22 | 22 (100.0) |
| Reticulation, no. (%) | 22 | 11 (50.0) |
| Bronchiectasis, no. (%) | 22 | 13 (59.1) |
| Lung micronodules, no (%) | 22 | 7 (31.8) |
| Honeycombing, no. (%) | 22 | 5 (22.7) |
| Goh classification ^†^, limited, no. (%) | 22 | 9 (40.9) |
| Lung fibrosis ^†^, median (IQR), % | 22 | 50.0 (10.0; 80.0) |
| **Pulmonary function tests** |  |  |
| Duration from PET, median (IQR), months | 22 | 0.0 (-1.0; 1.0) |

ILD: interstitial lung disease; SSc: systemic sclerosis. ^†^ lung fibrosis extent estimated on HRCT scan according to Goh et al(36)
