# Supplementary material for: 18F-FDG positron emission tomography scanning in systemic sclerosis-associated interstitial lung disease: a pilot study
Source: Arthritis Res Ther. 2021 Mar 6;23:76. doi: 10.1186/s13075-021-02460-8 (PMC7936499; doi:10.1186/s13075-021-02460-8)
Supplement: Supplementary file 2 — Additional file 2 Correlation between FDG PET/CT scan findings, lung fibrosis extent (%), %DLCO and %FVC (n = 36). [file 13075_2021_2460_MOESM2_ESM.docx]

**Additional file 2.** Correlation between FDG PET/CT scan findings, lung fibrosis extent (%), %D_LCO_ and %FVC (n=36)

|  | **hv/SUV_max_** | | **S/SUV_max_** | | **S/Intensities** | | |
| --- | --- | --- | --- | --- | --- | --- | --- |
| **Correlation (Spearman)** | **Corr. Sp.** | **p value** | **Corr. Sp.** | **p value** | **Corr. Sp.** | **p value** |  |
| %Fibrosis extent ^†^ | 0.342 | 0.12 | 0.475 | 0.026 | 0.347 | 0.11 |  |
| %FVC | -0.173 | 0.33 | -0.308 | 0.076 | -0.426 | 0.012 |  |
| %D_LCO_ | -0.438 | 0.011 | -0.494 | 0.004 | -0.484 | 0.004 |  |

%FVC: forced vital capacity (% of predicted value); %D_LCO_: diffusing capacity for the lung of carbon monoxide; ^†^ lung fibrosis extent (%) assessed on HRCT according to Goh’s staging(36). For this parameter, correlation was assessed only in ILD-SSc (n=22)(31)
