# Supplementary material for: 18F-FDG positron emission tomography scanning in systemic sclerosis-associated interstitial lung disease: a pilot study
Source: Arthritis Res Ther. 2021 Mar 6;23:76. doi: 10.1186/s13075-021-02460-8 (PMC7936499; doi:10.1186/s13075-021-02460-8)
Supplement: Supplementary file 3 — Additional file 3 Characteristics and FDG PET/CT scan findings in SSc patients with limited ILD (n = 9) or with extensive ILD (n = 13). [file 13075_2021_2460_MOESM3_ESM.docx]

**Additional file 3.** Characteristics and FDG PET/CT scan findings in SSc patients with limited ILD (n=9) or with extensive ILD (n=13)

|  | **SSc Patients with limited ILD**  **(n=9)** | **SSc Patients with extensive ILD**  **(n=13)** |
| --- | --- | --- |
| **Demographics** |  |  |
| Sex, female, no. (%) | 6 (66.7) | 4 (30.8) |
| Age, years, mean ±SD | 54.7 ±14.5 | 63.9 ±8.7 |
| BMI, kg/m^2^, median (IQR) | 24.1 (21.4; 27.7) | 23.6 (22.1; 24.7) |
| **Disease characteristics** |  |  |
| Cutaneous subset, limited, no. (%) | 5 (55.6) | 6 (46.2) |
| Disease duration, <2years, no. (%) | 2 (22.2) | 4 (30.8) |
| Antibody status, no. (%) |  |  |
| Anti-centromere | 3 (33.3) | 2 (15.4) |
| Anti-topoisomerase I | 2 (22.2) | 7 (53.9) |
| Anti-RNA polymerase III | 2 (22.2) | 1 (7.7) |
| **Organ involvement** |  |  |
| Current mRSS, median (IQR) | 12.0 (6.0; 14.0) | 12.0 (2.0; 27.0) |
| Lung |  |  |
| Current %FVC, mean ±SD | 98 ±13 | 77 ±22 |
| Current %D_LCO_, mean ±SD | 58 ±18 | 45 ±20 |
| Lung fibrosis ^†^, %, median (IQR) | 9 (6; 20) | 74 (61; 83) |
| **PET parameters** |  |  |
| Hilar and/or mediastinal lymph nodes |  |  |
| Abnormal pattern, no. (%) | 2 (22.2) | 7 (53.9) |
| Lung |  |  |
| Abnormal pattern, no. (%) | 3 (33.3) | 11 (84.6) |
| hv/SUV_max_, mean ±SD | 2.3 ±0.9 | 2.8 ±0.9 |
| S/SUV_max_, mean ±SD | 14.9 ±4.4 | 18.9 ±6.5 |
| S/Intensities, median (IQR) | 6.0 (0.0; 8.0) | 10 (6.0; 13.0) |

%D_LCO_: diffusing capacity for the lung of carbon monoxide (% predicted value); %FVC: forced vital capacity (% predicted value); hv/SUV_max_: highest value of the SUV_max_ among the 10 pulmonary SUV_max_; IQR: interquartile range; SD: standard deviation; S/SUV_max_: sum of the 10 pulmonary SUV_max_; S/Intensities: Sum of the 10 pulmonary intensities; ^†^ lung fibrosis extent (%) on HRCT scan according to Goh’s staging(36).
