# Supplementary material for: 18F-FDG positron emission tomography scanning in systemic sclerosis-associated interstitial lung disease: a pilot study
Source: Arthritis Res Ther. 2021 Mar 6;23:76. doi: 10.1186/s13075-021-02460-8 (PMC7936499; doi:10.1186/s13075-021-02460-8)
Supplement: Supplementary file 4 — Additional file 4. Characteristics and FDG PET/CT scan findings in SSc patients with stable ILD (n = 13) or progressing ILD (n = 9) within 2 years after PET/CT. [file 13075_2021_2460_MOESM4_ESM.docx]

**Additional file 4.** Characteristics and FDG PET/CT scan findings in SSc patients with stable ILD (n=13) or progressing ILD (n=9) within 2 years after of PET/CT

|  | **SSc Patients with stable ILD**  **(n=13)** | **SSc Patients with progressing ILD**  **(n=9)** |
| --- | --- | --- |
| **Demographics** |  |  |
| Sex, female, no. (%) | 7 (53.9) | 3 (33.3) |
| Age, years, mean ±SD | 56.2 ±12.1 | 65.8 ±9.8 |
| BMI, kg/m^2^, median (IQR) | 24.1 (21.4; 27.7) | 23.3 (22.1; 24.6) |
| **Disease characteristics** |  |  |
| Cutaneous subset, limited, no. (%) | 6 (46.2) | 5 (55.6) |
| Disease duration, <2years, no. (%) | 2 (15.4) | 4 (44.4) |
| Antibody status, no. (%) |  |  |
| Anti-centromere | 4 (30.8) | 1 (11.1) |
| Anti-topoisomerase I | 4 (30.8) | 5 (55.6) |
| Anti-RNA polymerase III | 3 (23.1) | 0 (00.0) |
| **Organ involvement** |  |  |
| Current mRSS, median (IQR) | 12.0 (4.0; 20.0) | 12.5 (5.0; 21.0) |
| Lung |  |  |
| Current %FVC, mean ±SD | 92 ±17 | 77 ±22 |
| Current %D_LCO_, mean ±SD | 53 ±16 | 45 ±20 |
| Goh classification, limited, no. (%) | 7 (53.8) | 2 (22.2) |
| Lung fibrosis ^†^, %, median (IQR) | 20 (9; 50) | 70 (61; 77) |
| Changes of %FVC ^‡^, median (IQR) | +4 (+1; +8) | -11 (-6; -14) |
| Follow-up after PET/CT, months, median (IQR) | 22.9 (17.8; 29.0) | 25.6 (14.1; 27.0) |
| **PET parameters** |  |  |
| Hilar and/or mediastinal lymph nodes |  |  |
| Abnormal pattern, no. (%) | 4 (30.8) | 5 (55.6) |
| Lung |  |  |
| Abnormal pattern, no. (%) | 6 (46.2) | 8 (88.9) |
| hv/SUV_max_, mean ±SD | 2.4 ±1.0 | 2.8 ±0.6 |
| S/SUV_max_, mean ±SD | 14.5 ±5.0 | 21.3 ±5.0 |
| S/Intensities, median (IQR) | 3.0 (0.0; 8.0) | 12 (8.0; 13.0) |

%D_LCO_: diffusing capacity for the lung of carbon monoxide (% predicted value); %FVC: forced vital capacity (% predicted value); hv/SUV_max_: highest value of the SUV_max_ among the 10 pulmonary SUV_max_; IQR: interquartile range; SD: standard deviation; S/SUV_max_: sum of the 10 pulmonary SUV_max_; S/Intensities: Sum of the 10 pulmonary intensities; ^†^ lung fibrosis extent (%) on HRCT scan according to Goh’s staging(36); ^‡^ Changes of %FVC within the 2 years ±6months after PET/CT.
