# Supplementary material for: 18F-FDG positron emission tomography scanning in systemic sclerosis-associated interstitial lung disease: a pilot study
Source: Arthritis Res Ther. 2021 Mar 6;23:76. doi: 10.1186/s13075-021-02460-8 (PMC7936499; doi:10.1186/s13075-021-02460-8)
Supplement: Supplementary file 5 — Additional file 5 Additional FDG PET/CT scan findings in controls (n = 89) and in SSc patients (n = 36). [file 13075_2021_2460_MOESM5_ESM.docx]

**Additional file 5**. Additional FDG PET/CT scan findings in controls (n=89) and in SSc patients (n=36)

|  | **Controls**  (n = 89) | **SSc Patients**  (n = 36) |
| --- | --- | --- |
| **Muscle, mean SUV_max_ ±SD** |  |  |
| Right deltoid | 1.3 ±0.3 | 1.3 ±0.3 |
| Left deltoid | 1.3 ±0.3 | 1.2 ±0.3 |
| Right pectoral | 1.1 ±0.2 | 1.1 ±0.3 |
| Left pectoral | 1.3 ±0.3 | 1.3 ±0.2 |
| Right rectus abdominus | 1.1 ±0.3 | 1.2 ±0.5 |
| Left rectus abdominus | 1.1 ±0.3 | 1.1 ±0.3 |
| Right quadriceps | 1.3 ±0.4 | 1.3 ±0.4 |
| Left quadriceps | 1.3 ±0.3 | 1.3 ±0.4 |
| **Skin, mean SUV_max_ ±SD** |  |  |
| Right shoulder | 0.7 ±0.2 | 0.7 ±0.2 |
| Left shoulder | 0.7 ±0.2 | 0.7 ±0.2 |
| Right breast | 0.8 ±0.3 | 0.9 ±0.3 |
| Left breast | 0.8 ±0.3 | 0.8 ±0.3 |
| Peri-umbilic | 0.9 ±0.4 | 1.0 ±0.4 |
| Right thigh | 0.8 ±0.2 | 0.9 ±0.3 |
| Left thigh | 0.7 ±0.2 | 0.8 ±0.3 |
| **Osteoarticular, mean SUV_max_ ±SD** |  |  |
| Right gleno-humeral | 2.6 ±0.5 | 2.8 ±0.5 |
| Left gleno-humeral | 2.6 ±0.4 | 2.4 ±0.4 |
| Right coxo-femoral | 2.9 ±0.7 | 2.9 ±0.7 |
| Left coxo-femoral | 2.9 ±0.6 | 3.1 ±1.1 |
| **Medulla, median SUV_max_ ±SD** |  |  |
| Rachis medulla | 4.0 ±1.2 | 3.7 ±1.1 |

SD: standard derivation; SSc: systemic sclerosis. ^†^ among patients with abnormal esophagus pattern of FDG uptake
