# Supplementary material for: 18F-FDG positron emission tomography scanning in systemic sclerosis-associated interstitial lung disease: a pilot study
Source: Arthritis Res Ther. 2021 Mar 6;23:76. doi: 10.1186/s13075-021-02460-8 (PMC7936499; doi:10.1186/s13075-021-02460-8)
Supplement: Supplementary file 6 — Additional file 6 Additional FDG PET/CT scan findings in SSc patients without ILD (n = 14) and those with ILD (n = 22). [file 13075_2021_2460_MOESM6_ESM.docx]

**Additional file 6.** Additional FDG PET/CT scan findings in SSc patients without ILD (n=14) and with ILD (n=22)

|  | **SSc Patients without ILD (n=14)** | **SSc Patients with ILD (n=22)** |
| --- | --- | --- |
| **Muscle, median SUV_max_ (IQR)** |  |  |
| Right deltoid | 1.3 (1.1; 1.5) | 1.2 (1.1; 1.4) |
| Left deltoid | 1.3 (1.1; 1.4) | 1.2 (1.0; 1.3) |
| Right pectoral | 1.2 (1.0; 1.4) | 1.0 (0.9; 1.2) |
| Left pectoral | 1.2 (1.0; 1.4) | 1.2 (0.9; 1.2) |
| Right rectus abdominus | 1.1 (1.0; 1.2) | 1.1 (0.9; 1.4) |
| Left rectus abdominus | 1.1 (1.0; 1.2) | 1.1 (1.0; 1.3) |
| Right quadriceps | 1.3 (1.0; 1.6) | 1.1 (1.0; 1.6) |
| Left quadriceps | 1.3 (1.1; 1.6) | 1.3 (1.0; 1.5) |
| **Skin, median SUV_max_ (IQR)** |  |  |
| Right shoulder | 0.7 (0.5; 0.7) | 0.8 (0.6; 0.9) |
| Left shoulder | 0.6 (0.5; 0.9) | 0.8 (0.6; 0.9) |
| Right breast | 0.9 (0.7; 1.1) | 0.9 (0.6; 1.1) |
| Left breast | 0.8 (0.7; 1.1) | 0.8 (0.6; 0.9) |
| Peri-umbilic | 0.8 (0.5; 1.0) | 1.0 (0.8; 1.3) |
| Right thigh | 0.9 (0.7; 1.0) | 0.8 (0.6; 1.0) |
| Left thigh | 0.8 (0.6; 1.0) | 0.8 (0.6; 1.0) |
| **Medulla, median SUV_max_ (IQR)** |  |  |
| Rachis medulla | 3.7 (2.6; 4.3) | 3.4 (2.7; 4.3) |

ILD: interstitial lung disease; IQR: interquartile range; SSc: systemic sclerosis
