# Supplementary material for: 18F-FDG positron emission tomography scanning in systemic sclerosis-associated interstitial lung disease: a pilot study
Source: Arthritis Res Ther. 2021 Mar 6;23:76. doi: 10.1186/s13075-021-02460-8 (PMC7936499; doi:10.1186/s13075-021-02460-8)
Supplement: Supplementary file 7 — Additional file 7. Visual examination of FDG PET/CT scans of a case report. [file 13075_2021_2460_MOESM7_ESM.docx]

**Additional file 7.** Visual examination of FDG PET/CT scans of a case report.

A dcSSc patient with anti-RNA polymerase III antibodies (mRSS=30/51) who had an abnormal interstitial lung pattern of FDG uptake without ILD on HRCT at inclusion. This patient developed later an SSc-ILD.


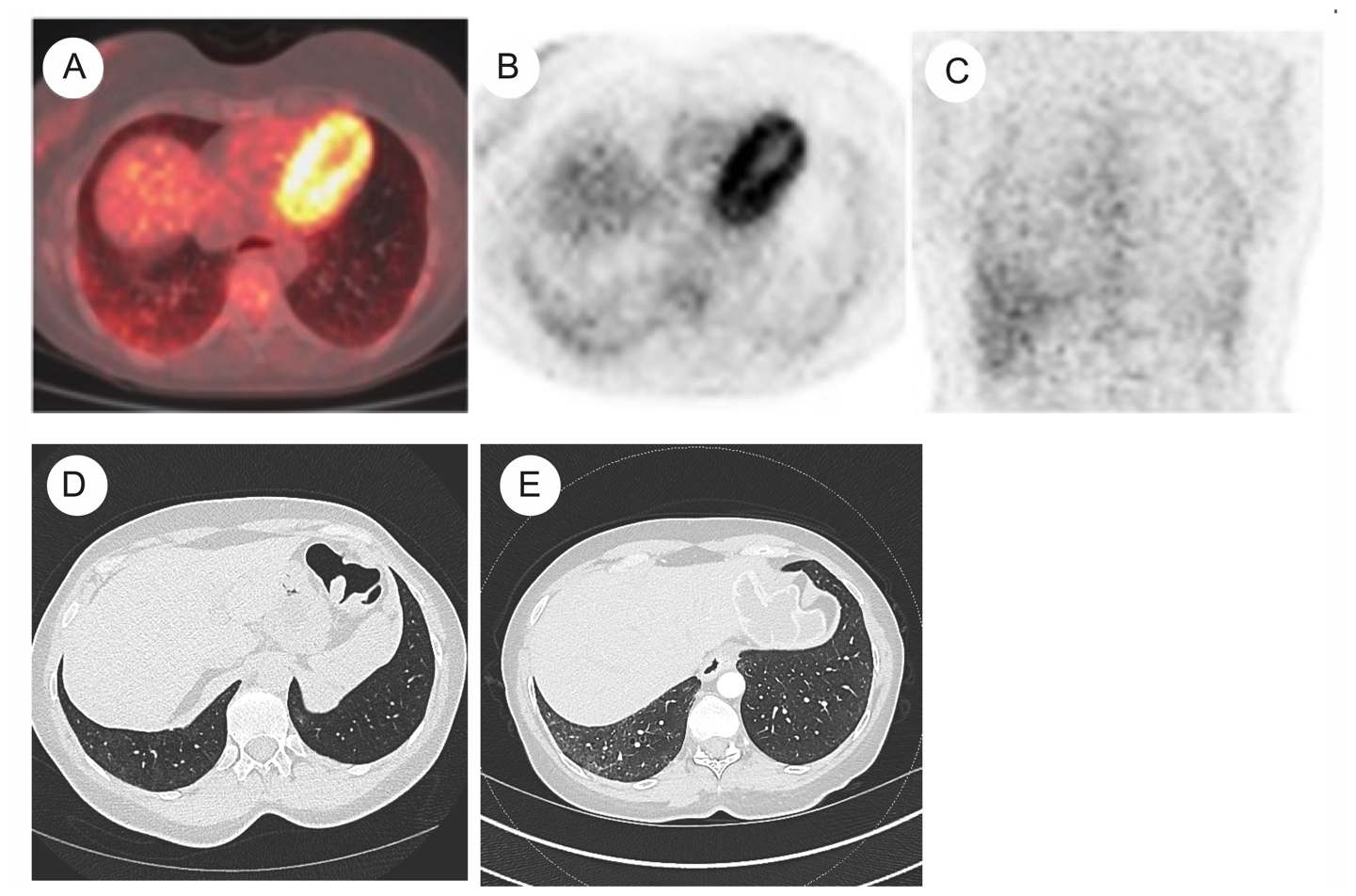


A to D: Transverse slides of the FDG PET/CT scans (A to C) and HRCT (D) scans showing an abnormal interstitial lung pattern without SSc-ILD at inclusion. E: HRCT scans 18 months later showing an SSc-ILD.
